# Australian graduating nurses’ knowledge, intentions and beliefs on infection prevention and control: a cross-sectional study

**DOI:** 10.1186/s12912-014-0043-9

**Published:** 2014-12-12

**Authors:** Brett G Mitchell, Richard Say, Anne Wells, Fiona Wilson, Linda Cloete, Lucinda Matheson

**Affiliations:** Faculty of Nursing and Health, Avondale College of Higher Education, Wahroonga, NSW 2076 Australia; School of Nursing, Midwifery and Paramedicine, Australian Catholic University, Dickson, ACT Australia; School of Health Sciences, University of Tasmania, Hobart, TAS 7005 Australia; Department of Health and Human Services, Tasmanian Infection Prevention and Control Unit, Hobart, Tasmania Australia

## Abstract

**Background:**

In recent year, national bodies have been actively addressing the increasing concern on the spread of healthcare-associated infections (HAIs). The current study measures the knowledge, intentions and beliefs of third-year Australian nursing students on key infection prevention and control (IPC) concepts.

**Methods:**

A cross-sectional study of final-year undergraduate nursing students from Schools of Nursing at six Australian universities was undertaken. Students were asked to participate in an anonymous survey. The survey explored knowledge of standard precautions and transmission based precautions. In addition intentions and beliefs towards IPC were explored.

**Results:**

349 students from six universities completed the study. 59.8% (95% CI 58.8–60.8%) of questions were answered correctly. Significantly more standard precaution questions were correctly answered than transmission-based precaution questions (p < 0.001). No association was found between self-reported compliance with IPC activities and gender or age. Certain infection control issues were correlated with the percentage of correctly answered transmission-based precaution questions. The participants were most likely to seek infection control information from an infection control professional.

**Conclusion:**

Knowledge on transmission-based precautions was substandard. As transmission-based precautions are the foundation of IPC for serious organisms and infections, education institutions should reflect on the content and style of educational delivery on this topic.

**Electronic supplementary material:**

The online version of this article (doi:10.1186/s12912-014-0043-9) contains supplementary material, which is available to authorized users.

## Background

In recent years, there has been an effort by organisations internationally to reduce the spread of healthcare-associated infections (HAIs). Healthcare associated infection is the contemporary term used to refer to infections acquired in healthcare facilities and infections that occur as a result of healthcare interventions [1]. The effect of HAIs are not only felt by individual patients through increased morbidity and mortality, but also by a health service through higher costs associated with infections. The magnitude of the effect of HAIs on patients is evidenced by a report from the World Health Organisation, which suggests that 7.6% of hospitalised patients will acquire a HAI [2]. This is support by other point prevalence studies who have found similar incidence of infections [3-5]. In response to the prevention of HAIs in Australia, the Australian Commission on Safety and Quality in Health Care (ACSQHC) launched the *Healthcare Acquired Infection Prevention Program* in 2009 [6], and the National Health and Medical Research Council (NHMRC) released the *Australian Guidelines for the Prevention and Control of Infection in Healthcare* in 2010 [1].

The ACSQHC developed initiatives which aim to standardise infection prevention and control (IPC) practice across Australia as a part of a wide strategy to reduce the incidence of HAIs. However, in the approach to reducing HAIs in Australia, there has been little mention of the role of undergraduate education. This is despite evidence pointing towards the pivotal role of the undergraduate environment in shaping attitude and building the knowledge of healthcare professionals [7].

The prevention of infection requires a multifaceted approach. Infectious agents can be transmitted from their sources to a susceptible host by a variety of methods, which include direct spread on the hands of healthcare workers, carriage on equipment, and through airflow. The prevention of transmission via these methods can be routinely prevented in healthcare settings using ‘standard precautions’. Standard precautions include hand hygiene and the use of personal protection equipment when anticipating contact with blood or bodily fluids. Contact precautions are applied to the care of all patients at all times [1,8]. Other modes of transmission for infectious agents are via direct or indirect contact, mucous membrane contact with respiratory secretions (droplet transmission) or inhalation of infectious agents suspected in the air (airborne transmission). These three processes fall under the umbrella term ‘transmission based precautions’ [1,8].

Despite the well documented use of standard and transmission based precautions to prevent infection, there is a lack of conceptual and theoretical frameworks in the field of IPC [9]. None the less, the first step in informing IPC education in undergraduate curricula is to determine a baseline, described as surveillance in a theoretical framework proposed by Mitchell and Gardner [10]. The current study measures the knowledge, intentions and beliefs of third-year Australian nursing students on key infection prevention and control (IPC).

Clinician education is a key strategy in the reduction of HAIs through IPC programs. The ACSQHC names education as a central tenet in effective IPC strategies [6,11]. Overseas, the United States (US) Department of Health and Human Services highlights the critical role of education in IPC, and numerous National Health Service trusts and government departments in the United Kingdom (UK) emphasise the importance of education in IPC [12,13]. Despite local and international acknowledgement that education is key, however, concerns surrounding deficits in IPC knowledge amongst clinicians, including attitude towards IPC, continue to be reported [14-16]. Knowledge is a precursor to changing entrenched attitude, and numerous studies have emphasised the importance of both of these components for a sustainable behavioural change amongst clinicians [17]. This relationship among knowledge, attitude and behavioural change has been specifically researched in relation to IPC [14,18].

Pre-registration training plays a crucial role in improved clinician compliance to IPC [7], and the NHMRC asserts that undergraduate clinician programs serve as a key environment where knowledge acquisition on IPC should occur [1]. A number of studies highlight the inadequacy of many undergraduate programs to effectively train students of various clinical disciplines [7,13,19-22]. This finding implies the possible causal link between undergraduate education and the lack of clinician compliance to IPC policy.

A number of authors have pointed out the lack of research which examines the effectiveness of IPC programmes in undergraduate nursing curricula [13,19,23]. This case is particularly notable in Australia. Nurses represent the largest healthcare service labour group which has a high frequency of contact with patients [24], so IPC education of nurses in undergraduate programmes warrants attention. To the best of our knowledge, no study which examines undergraduate nursing IPC programmes in Australia has been undertaken.

The data collected in this study measured IPC knowledge, intentions and beliefs of final-year nursing students across six universities in Australia. We believe that this research can act as a starting point to inform undergraduate curricula in Australia on effective IPC training.

## Methods

### Aim

This study aims to determine graduating nursing students’ knowledge of and intentions towards IPC practices. The following research questions which underpin this project are directed to graduating nursing students:

1. What is their knowledge of standard precautions and its application?
2. What is their knowledge of transmission-based precautions and its application?
3. How and where do they seek information about IPC activities?
4. What is their belief of infection risk for IPC activities?

### Design

A cross-sectional study was used in this research.

### Setting and participants

The participants in this study were final-year nursing bachelorette degree students at six universities in Australia. The students were surveyed at the end of their final year of study, with their graduation anticipated to occur in the following three months.

### Data collection

The department heads of the nursing and midwifery schools of eight universities were contacted, and access to their final-year nursing students was requested. Six schools agreed to participate and forwarded an invitation e-mail to their students. In the invitation, the students were asked to participate in an anonymous survey, with no obligation to participate. To improve the response rate, we offered an incentive of winning an Ipad to prospective respondents.

An online survey was developed with the help of the research team, trialled with infection control experts and cross-referenced against national guidelines to determine the correct response [1]. The survey asked basic demographic information, including age, sex, education institution and anticipated data of graduation. A measure of academic performance, such as a grade point average was not collected – the pilot of the survey indicated a lack of awareness of this and inconsistency in application.

The questions contained in the survey consisted of ten multiple choice, seven true/false and questions that required the student to rank responses. The survey topics explored the respondents’ knowledge and application of standard and transmission-based precautions, as well as built on previous studies and existing tools [16,25,26] (Additional file 1). Questions relating to hand hygiene were taken from an online competency packaged developed by Hand Hygiene Australia [27]. This package has been extensively used by nurses in the Australian hospital system. The survey was piloted on second year-nursing students and subsequently revised on the basis of feedback from the pilot.

### Ethical considerations

The study was approved by the Avondale College for Higher Education Human Research Ethics Committee (2013:22). All participants provided consent to participate.

### Statistical analysis

**Figure 1:**
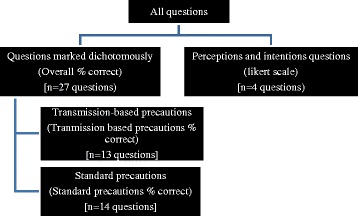
**Overview of data management for analysis.**

Data were analysed with IBM Statistic SPSS (SPSS, Chicago, IL, USA) [28]. The responses to questions used in the survey were categorised into two groups: questions which can be marked in a dichotomous manner (i.e., correct or incorrect) and questions which explored intentions and beliefs. An overall percentage score was calculated for correctly answered questions. In addition, the questions were further divided into two themes: standard precaution questions and transmission-based precaution questions (Figure 1).

For proportions, 95% confidence intervals (CI) were calculated for Poisson distributed counts. Independent t-tests were performed to compare two variables mean scores. Comparisons of non-parametric independent demographic data were conducted with the use of the Mann–Whitney test. For questions which required the participants to rank a response, mean scores and standard deviations were calculated. Analysis of variance (ANOVA) was used to compare any differences between variable mean scores. Correlations between variables were calculated with Spearman’s correlation coefficient or Kendall’s tau for non-parametric correlations.

## Results

### Overview

A total of 349 students from six universities completed the study. The response rate represents 21% of all graduating student nurses from these institutions. The age of the participants ranged from 19 to 65 years, with a median of 25 years. Of the total respondents, 319 (91.4%) were female. The percentage of questions correctly answered was 59.8% (95% CI 58.8–60.8%). Table 1 summarises the mean weighted score by demographic. Notably, significantly more standard precaution questions were correctly answered than transmission-based precaution questions (p < 0.001). The mean score for the standard and transmission-based precaution questions did not vary between age groups or sex. Table 1 provides a summary of the correct answers by demographic.

**Table 1:** **Percentage of questions answered correctly**

**Demographic**	**Percentage answered correctly (mean)**	**95% CI**	**SD**	**p value**
Age group (IQR)				
19-21 years (n = 83)	60.0%	57.9 -62.1	9.8	
22-25 years (n = 87)	60.1%	58.3 -62.0	8.6	
26-35 years (n = 81)	60.3%	58.2 -62.5	9.7	
>35 years (n = 98)	58.9%	57.1 -60.7	9.4	0.74
Sex				
Male (n = 30)	59.7%	58.7 -60.8	9.4	0.68
Female (n = 319)	60.4%	57.1 -63.6	8.8	
Question theme				
Standard precautions (n = 349)	88.9%	88.1-89.8	8.4	<0.01
Transmission-based precautions (n = 338)	27.2%	25.6-28.8	14.7	
Total (n = 349)	59.8%	58.8 -60.8	9.3	

Note: SD = Standard deviation. IQR = interquartile range. 95% CI = 95% confidence interval.

### Compliance

When asked to assess their compliance with a range of IPC activities, the majority of participants indicated that their behaviour was consistent with the best practice, as summarised in Table 2. No association was found between self-reported compliance with IPC activities and gender or age. However, a correlation was identified between the percentage of correctly answered standard precaution questions and the participants’ self-reported compliance with three standard precaution activities (Table 3).

**Table 2:** **Self-reported compliance against infection control procedures (n = 338)**

**Statement**	**Always (n)**	**Mostly (n)**	**Occasionally (n)**	**Rarely (n)**	**Never (n)**
I use gloves when I anticipate exposure to blood or bodily fluid	96% (323)	4% (14)	0% (1)	-	-
I change gloves between patients	96% (326)	4% (10)	0% (1)	0% (1)	-
I clean medical equipment after use	52% (177)	37% (126)	9% (29)	1% (4)	1% (2)
I RECAP needles after giving an injection	3% (12)	1% (2)	3% (10)	11% (37)	82% (277)
I wear eye protection when I am at risk of blood or body fluid splashes to my eyes	63% (214)	26% (88)	6% (20)	4% (14)	1% (2)
I perform hand hygiene before I touch a patient	82% (276)	17% (59)	1% (3)	-	-
I educate, encourage and assist (if needed) my patients to perform hand hygiene after going to the toilet and before eating.	57% (192)	28% (95)	10% (33)	3% (12)	2% (6)

**Table 3:** **Correlations between self-reported compliance infection prevention and control activities and the percentage of correctly answered questions (N = 349)**

	**Percentage correctly answered SBP questions**		**Percentage correctly answered TBP questions**		**Percentage correctly answered all questions**	
**Variables**	**Correlation Coefficient**	**p value**	**Correlation Coefficient**	**p value**	**Correlation Coefficient**	**p value**
I use gloves when I anticipate exposure to blood or bodily fluid	0.113	0.02	0.006	0.90	0.068	0.15
I change gloves between patients	0.137	0.01	0.004	0.93	0.089	0.06
I clean medical equipment after use	0.016	0.74	−0.090	0.05	−0.062	0.17
I recap needles after giving an injection	−0.006	0.91	0.010	0.84	0.000	0.99
I wear eye protection when I am at risk of blood or body fluid splashes to my eyes	0.067	0.17	−0.088	0.06	−0.031	0.49
I perform hand hygiene before I touch a patient	0.101	0.04	−0.087	0.07	−0.012	0.80
I educate, encourage and assist (if needed) my patients to perform hand hygiene after going to the toilet and before eating	0.004	0.93	−0.088	0.05	−0.061	0.18

Note: SBP = Standard precautions, TBP = Transmission-based precautions.

### Beliefs

**Figure 2:**
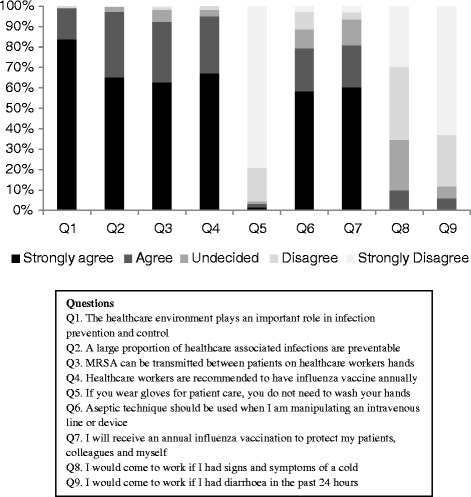
**Students’ beliefs regarding infection control issues.**

Figure 2 shows the students’ beliefs towards a range IPC concepts. Approximately 60% of the students strongly agreed that a large proportion of HAIs were preventable, MRSA can be transmitted via healthcare workers’ hands, aseptic technique should be used in manipulating intravascular devices and that they will receive an annual influenza vaccination. When the respondents were asked whether they will still go to work if they have a cold, 10% said they will, 25% were undecided and 65% said they will not.

The participants held similar views on whether a range of topics posed an infection control problem in Australian hospitals. On a scale of one to four, with one being not a problem and four being a serious problem, the participants rated MRSA (*×* 3.76, SD 0.46), *Clostridium difficile* infection (*×* 3.52, SD 0.65), multi-resistant Gram negative organisms (*×* 3.60, SD 0.57), antibiotic resistance (*×* 3.64, SD 0.59), low levels of hand hygiene compliance (*×* 3.41, SD 0.76) and gastroenteritis outbreaks *(×* 3.48, SD 0.64) as problems. However, the participants viewed low levels of environmental cleanliness (× 3.10, SD 0.88), blood stream infections (*×* 3.13, SD 0.80), urinary tract infections (*×* 3.22, SD 0.81) and needle stick injuries (× 3.22, SD 0.80) as lesser problems than the previously stated issues. This difference was significant at the 0.05 level. The participants’ views on these topics were correlated with the percentage of correctly answered transmission-based precaution questions. As the percentage of correctly answered transmission-based precaution questions increased, the concern on gastroenterological outbreaks decreased (r = −0.128, p = 0.007). Conversely, an increased concern on antimicrobial resistance was correlated with an increase in the total percentage of questions correctly answered (r = 0.093, p = 0.049).

### Information seeking behaviour

The participants were most likely to seek IPC information from an infection control professional (× 1.82, SD 0.79), followed by organisational policies and guidelines (× 1.89, SD 0.79). They were significantly less likely to seek information from senior nurses, scientific journals and the Internet compared with consulting infection control professionals (p < 0.001) and hospital policies (p < 0.001). As regards information seeking behaviour by age group, those aged less than 25 years were more likely to seek information from senior nurses (p = 0.02) and the Internet (p = 0.02) than those aged 25 years and older. Conversely, those aged 25 years and older were more likely to seek information from organisational policies than those aged less than 25 years (p = 0.02).

## Discussion

The nursing students in this study demonstrated inadequate knowledge on IPC, more specifically transmission based precautions, a finding consistent with those of previous studies [13,21,23]. The participants demonstrated a considerably stronger level of knowledge on the topic of standard precautions than transmission-based precautions, with 88.9% of standard precaution questions correctly answered. The literature indicated mixed results in relation to this theme. Undergraduate healthcare students were found to have an acceptable level of standard precaution knowledge in some studies [19,25,29] and knowledge deficit in others [8]. The low level of knowledge in relation to transmission-based precautions observed in our study is consistent with the findings in the literature [19,30]. We identified only 27.2% of transmission-based precaution questions correctly answered. This result suggests that undergraduate education on transmission-based precautions may be inadequate; this observation is particularly pertinent because of the increasing presence of new and evolving pathogens in healthcare environments and the effect of clinician education on reducing the spread of HAIs [11].

We found negative correlations between the participants’ increased knowledge of transmission-based precautions and whether they thought specific issues posed an IPC problem in Australian hospitals. Neither gastroenteritis outbreaks, nor hand hygiene compliance were considered to pose a problem. We postulate that a possible reason for this finding is the respondents’ experiences and exposure to these issues during their undergraduate education and more specifically, during their clinical placements. For example, graduating nurses may have had little or no nursing experience of a gastroenteritis outbreak. Unless a student nurse has worked in a healthcare setting during an outbreak, he or she may not consider outbreaks as an IPC issue. Furthermore, the participants’ view that hand hygiene compliance was less of a problem relative to other scenarios posed can be explained by the popularity of hand hygiene in Australia. The National Hand Hygiene Initiative was introduced across Australia in 2009. This major initiative and its subsequent publicity may have led to the perception that hand hygiene is no longer an issue in healthcare.

Our study also identified some interesting themes on how student nurses obtain IPC information. Several well-reported international studies suggest that nurses prefer to approach colleagues for information rather than access evidence-based resources [31-35]. This pattern of information-seeking behaviour is also evident in graduate and undergraduate nurses, primary health nurses, and acute and critical care nurses [36-38]. As an example, three quarters of the nurses studied by O’leary and Mhaolŕunaigh [35] consulted human sources of information on a daily or weekly basis. The literature also reveals that nurses generally ask for information from a colleague perceived to be more knowledgeable or experienced than they are, whereas they considered text-based resources as useful only when these are readily available [35,37]. The fact that the participants in our study were more likely to seek information from an infection control professional is supported by the themes previously discussed and is consistent with the information-seeking behaviour amongst all grades of nurses.

The participants in our study, aged 25 years or younger, were more likely to seek information from senior nurses, compared with those participants who were over 25 years. This finding is consistent with that of an Irish study, which found that compared with more experienced nurses, less experienced ones were reported to more heavily rely on other people for information [35]. Similarly, a UK study found that other staff were likely to influence student nurses’ IPC practice [13]. As nurses gain experience, they are more likely to seek information from the next most available information source, for example, policies and guidelines [35]. The reasons cited in the literature for consulting human sources instead of evidence-based information include convenience and efficiency, a perceived lack of computer skills, and avoidance of large amounts of retrieved information which are still to be read, analysed and evaluated [37,38]. O’Leary and Mhaolrunaigh [35] suggest that nurses only search for a limited time to find information which they self-determine to be an acceptable solution and that they do not search long enough to find a solution which they can consider as the ‘best decision’. Evidence-based information is therefore recommended to be relevant for practice and must be made readily available [35]. Furthermore, undergraduate nursing curricula should be designed in such a way that they develop the necessary skills for students to understand the importance of and ability to access evidence-based practice.

### Limitations

Our study involves limitations. A cross-sectional web based survey is not an ideal marker of practice. For this reason, our study focused on knowledge, intentions and beliefs rather than self-reported practice. How knowledge is translated into practice by the participants of our study remains unknown. Furthermore, our study surveyed student nurses from different universities which expectedly have different curricula, so the timing of IPC education or skills training also differs. This variation was not properly accounted for in our study. The potential of selection bias also exists in our research. The students were invited via e-mail to participate in the study, and they then decided whether to join. The motivation to participate and whether the motivation was related to academic performance are unknown. Finally, the response rate a response rate of 21% was achieved for this study, therefore the generalisability of the findings needs to be undertaken with caution.

## Conclusion

We found that in the final phase of their undergraduate education, Australian student nurses had adequate levels of knowledge on standard precautions principles of IPC. However, their knowledge on transmission-based precautions was substandard. Transmission-based precautions are the foundation of IPC for serious organisms and infections, so several implications can be determined. Those responsible for developing undergraduate nursing curricula should consider whether the current approach to IPC used in their respective institutions is effective. For organisations which employ nursing graduates, whether additional IPC training is required should also be seriously considered. Infection prevention and control professionals are often responsible for delivering education in hospitals, and the findings from our study can help them understand the current knowledge gaps in newly qualified nurses to allow for targeted education.

## Additional file

Additional file 1:
**Survey.**
