# Supplementary material for: Australian graduating nurses’ knowledge, intentions and beliefs on infection prevention and control: a cross-sectional study
Source: BMC Nurs. 2014 Dec 12;13:43. doi: 10.1186/s12912-014-0043-9 (PMC4266973; doi:10.1186/s12912-014-0043-9)
Supplement: Additional file 1: — Survey. [file 12912_2014_43_MOESM1_ESM.pdf]

Graduate nurses knowledge of infection prevention and control

Participant information

Dear Participant,

You are invited to participate in a short study designed to examine the infection control knowledge of nursing graduates. It will involve the completion of an anonymous on-line survey.

Participation in this project is not expected to pose any risks beyond your normal work day.

If you agree to participate in this project, you will be asked to respond to a short on-line survey, which is estimated to take 15 minutes to complete. The survey will ask you questions about infection control and healthcare associated infections.

Your participation in this study is voluntary. The results will be collected anonymously and on-line, so it is not possible to separate your responses from the rest of the data after they are submitted.

Your responses to this survey will be confidential and will only be accessed by the research team. Results from this survey will be aggregated and no individual data will be reported. At the end of the survey, you will be invited to indicate if you wish to enter in the draw for a prize. Any contact information you provide will be stored separately from your survey responses and all data will be stored in an encrypted form to protect your privacy. Data from this study may also be used in future research or may be provided to other researchers but only in a form that does not identify participants.

Important - this survey is being used to evaluate and plan future interventions. Please complete this survey honestly and without assistance. We are not interested in individual scores.

Any questions you may have regarding this project should be directed to Dr Brett Mitchell, Avondale College, Faulty of Nursing and Health, NSW; email: brett.mitchell@avondale.edu.au; phone: 0458 352 162

This research project has been approved by the Avondale College of Higher Education Human Research Ethics Committee (HREC). Avondale requires that all participants are informed that if they have any complaint concerning the manner in which a research project is conducted it may be given to the researcher, or if an independent person is preferred, to Avondale’s HREC Secretary, Avondale College of Higher Education, PO Box 19, Cooranbong NSW 2265, or phone (02) 4980 2121 or fax (02) 4980 2117 or email: research.ethics@avondale.edu.au

Graduate nurses knowledge of infection prevention and control

Consent

By agreeing to participate in this study, you are agreeing that you:
• have read and understood the information provided in the Information to Participants section.
• have had any questions answered to your satisfaction.
• agree to participate in this on-line survey
• understand that once you have submitted your responses, these cannot be withdrawn.
• agree that research data collected for this study may be published or may be provided to other researchers in a form that does not identify you in any way.

*1. Please indicate your response to the statements above and consent to participate

◯ I do not agree and do not give my consent

◯ I agree and give my consent

Graduate nurses knowledge of infection prevention and control

Information about you and your place of study

2. Age

*3. Gender

- Female
- Male
- Intersex

*4. Institution

5. Campus (if applicable)

*6. Anticipated year of graduation (final grade, not graduation ceronomy)

*7. Anticipated month of graduation (final grade, not graduation ceronomy)

*8. Have you been offered a position of employment following graduation?

Offer of employment

*9. If offered a position following graduation, please indicate the workplace

- ◯ Hospital (public)
- ◯ Hospital (private)
- ◯ Residential or aged care facility
- ◯ Community setting
- ◯ GP practice
- ◯ Research
- ◯ Continuing study
- ◯ Not yet working or offered a position

Other (please specify)

*10. Have you received infection control education during an induction or orientation program in the past 3 months? Typically, this would be provided when starting a new job.

- ◯ No
- ◯ Yes

The next few questions are multiple choice. Please select the best answer for each question.

* 11. Standard Precautions should be used

- a) For patients with a known infection
- b) For patients with a suspected infection
- c) For all patients
- d) For patients who look like they may have an infectious disease
- e) For patient where there is a risk of transmitting an infection.

* 12. The ‘5 moments for Hand hygiene’ should be performed

- a) Before and after touching a patient, before and after a procedure
- b) Before and after touching a patient, before and after a procedure and after touching a patients' surroundings.
- c) Before and after touching a patient, before and after a procedure and after going to the toilet
- d) After touching a patient, before and after a procedure, after touching a patients' surroundings

* 13. What is Hand Hygiene?

- a) Decontaminating hands using an alcohol based hand rub
- b) Washing hands with soap and water
- c) Washing hands using water only
- d) A & B

* 14. Contact precautions requires the use of

- a) Gown and gloves
- b) Surgical mask
- c) P2 (N95 mask)
- d) A and B
- e) A and D

* 15. Airborne precautions requires the use of

- a) Gown and gloves
- b) Surgical mask
- c) P2 (N95 mask)
- d) A and B
- e) A and D

*** 16. A patient is admitted with chickenpox. The staff working on this day are working out who can look after the patient without a risk of contracting the disease. Which of the following staff can look after the patient without a risk of contracting the disease?**

- ◯ a) All staff except pregnant nurses
- ◯ b) All staff who have had chickenpox
- ◯ c) All staff who wear a mask
- ◯ d) All staff with no restrictions
- ◯ e) Alls taff who wear gloves and an apron/gown

*** 17. What personal protective equipment can be used to prevent a staff member being exposed to blood or bodily fluids?**

- ◯ a) Gloves, eye protection and +/- gown.
- ◯ b) Safety devices
- ◯ c) Gloves and eye protection
- ◯ d) Gloves
- ◯ e) Eye protection

*** 18. Which of the following statements is most accurate?**

- ◯ a) Stethoscopes must be decontaminated between each patient
- ◯ b) Wrist watches should be removed as part of transmission based precautions but not standard precautions
- ◯ c) Wearing a hair cap is a requirement of transmission based precautions
- ◯ d) Jewellery should be removed as it harbours resident flora
- ◯ e) All of the above are correct

*** 19. From the list below, tick ALL the components of standard precautions (you may more than one).**

- ☐ Hand hygiene
- ☐ The use of personal protective equipment
- ☐ The safe use and disposal of sharps;
- ☐ Routine environmental cleaning;
- ☐ Reprocessing of reusable medical equipment and instruments
- ☐ Respiratory hygiene and cough etiquette
- ☐ Aseptic technique (includnig aseptic non touch technique)
- ☐ Waste management
- ☐ Appropriate handling of linen.

*** 20. Transmission based precautions may include which of the following (you may choose more than one)**

- [ ] Continued implementation of standard precautions
- [ ] Appropriate use of PPE (including gloves, apron or gowns, surgical masks/ P2 respirators and protective eyewear)
- [ ] Patient-dedicated equipment
- [ ] Allocation of single rooms or cohorting of patients
- [ ] Appropriate air handling requirements
- [ ] Enhanced cleaning and disinfecting of the patient environment
- [ ] Restricted transfer of patients within and between facilities

*** 21. Please indicate whether the following statements are true or false**

	True	False
Gloves must be worn as a single-use item for each invasive procedure, contact with sterile sites and non-intact skin or mucous membranes	○	○
Sterile gloves are not always required for aseptic procedures and contact with sterile sites	○	○
Frequently touched surfaces around a patient area (hospital) should be cleaned at least daily	○	○
Shared clinical equipment does not need to be cleaned between patient uses	○	○
Healthcare associated infections are a major patient safety issues, particularly in hospitals	○	○
The links in the chain of infection are all of the following: organism/pathogen, reservoir/source, portal of exit, mode of spread or tranmission, portal of entry, and susceptible host.	○	○
If you notice a hole in your gloves during a clinical procedure you should immediately remove your gloves and put on new gloves before continuing what you were doing. Hand hygiene is not required.	○	○

That is the end of the multiple choice questions. You now only have 6 more questions to complete.

*** 22. Tick all the types of precautions that could be applied when someone is placed under transmission based precautions (you can tick more than one answer).**

- [ ] Contact
- [ ] Droplet
- [ ] Airborne
- [ ] Faecal-oral
- [ ] Enteric

*** 23. For each of the patient scenarios below, tick all the measures you should take when providing routine nursing care to the patient such as assisting them with activities of daily living or taking routine observations**

	Standard precautions	Apron / Gown	Gloves	Surgical mask	P2 (N95 mask)	Eye protection
A patient who has MRSA in their nose	☐	☐	☐	☐	☐	☐
A patient who has pulmonary TB	☐	☐	☐	☐	☐	☐
A patient who has hepatitis B	☐	☐	☐	☐	☐	☐
A patient with a urinary tract infection and with an indwelling catheter	☐	☐	☐	☐	☐	☐
A person who has influenza	☐	☐	☐	☐	☐	☐

*** 24. Where would you seek information / advice about an infection control issue? (rank 1-5, 1 being the first place you would seek this information from)**

☐	Infection control professional in my organisation
☐	More senior nurses
☐	Scientific journals
☐	Organisation policies and procedures
☐	Internet

* 25. Please rank your compliance with the following activities

	Always	Mostly	Occasionally	Rarely	Never
I use gloves when I anticipate exposure to blood or bodily fluid	○	○	○	○	○
I change gloves between patients	○	○	○	○	○
I clean medical equipment after use	○	○	○	○	○
I RECAP needles after giving an injection	○	○	○	○	○
I wear eye protection when I am at risk of blood or body fluid splashes to my eyes	○	○	○	○	○
I perform hand hygiene before I touch a patient	○	○	○	○	○
I educate, encourage and assist (if needed) my patients to perform hand hygiene after going to the toilet and before eating.	○	○	○	○	○

* 26. Please rank your view on the following statements

	Strongly Agree	Agree	Undecided	Disagree	Strongly Disagree
The healthcare environment plays an important role in infection prevention and control	○	○	○	○	○
A large proportion of healthcare associated infections are preventable	○	○	○	○	○
MRSA can be transmitted between patients on healthcare workers hands	○	○	○	○	○
Healthcare workers are recommended to have influenza vaccine annually	○	○	○	○	○
If you wear gloves for patient care, you do not need to wash your hands	○	○	○	○	○
Aseptic technique should be used when I am manipulating an intravenous line or device	○	○	○	○	○
I will ensure I received an annual inflenza vaccination to protect my patients, colleagues and myself	○	○	○	○	○
I would come to work if I had signs and symptoms of a cold	○	○	○	○	○
I would come to work if I had diarrhoea in the past 24 hours, felt a little sick, but I was confident I could do my job well for that shift.	○	○	○	○	○

* 27. From the list of organisms, infections or topics below, please indicate whether you think these pose an infection control problem in Australian hospitals

	Not at all a problem	Minor problem	Moderate problem	Serious problem	Don't know
MRSA	○	○	○	○	○
Clostridium diffficile infection	○	○	○	○	○
Multi-resistance Gram negative organisms	○	○	○	○	○
Antibiotic resistance	○	○	○	○	○
Low standards of environmental cleanliness	○	○	○	○	○
Low hand hygiene compliance	○	○	○	○	○
Blood stream infection	○	○	○	○	○
Urinary tract infections	○	○	○	○	○
Gastro outbreaks	○	○	○	○	○
Needle stick injuries (sharps injury)	○	○	○	○	○
